# Comprehensive Profiling Reveals Sialyl‐Tn Upregulation and Prognostic Value in Prostate Cancer

**DOI:** 10.1111/pin.70140

**Published:** 2026-06-22

**Authors:** Kirsty Hodgson, Libby Blencoe, Erin Smith, Aswini Sasikumar, Ziqian Peng, Margarita Orozco‐Moreno, Richard Beatson, Paula A. Videira, Jennifer Munkley

**Affiliations:** ^1^ Newcastle University Centre for Cancer Newcastle University Institute of Biosciences Newcastle UK; ^2^ Comprehensive Cancer Centre King's College London London; ^3^ UCIBIO – Applied Molecular Biosciences Unit, Department of Life Sciences, NOVA School of Science and Technology | FCT NOVA Universidade NOVA de Lisboa Caparica Portugal; ^4^ Associate Laboratory i4HB ‐ Institute for Health and Bioeconomy, NOVA School of Science and Technology | FCT NOVA Universidade NOVA de Lisboa Caparica Portugal; ^5^ CDG & Allies – Professionals and Patient Associations International Network (CDG & Allies – PPAIN) Caparica Portugal

## Abstract

Prostate cancer is a common cancer in males and there is an urgent unmet clinical need to identify new therapies for advanced disease. Aberrant glycosylation is a hallmark of prostate cancer and plays a functional role in disease progression. The sialyl‐Tn antigen (sTn) has been widely studied in cancer, yet its involvement in prostate cancer remains relatively unexplored. Here, we utilise a novel anti‐sTn antibody (L2A5) to comprehensively monitor sTn expression levels in clinical prostate cancer tissues encompassing normal, benign, primary, metastatic castration‐resistant prostate cancer (CRPC), and in patient‐derived xenograft (PDX) tissues. We show that while sTn is detected at low or negligible levels in normal prostate tissues, it is expressed in 44% of prostate tumours, and prostate cancer patients with high sTn levels have significantly poorer survival times. Analysis of metastatic therapy‐resistant prostate‐derived tumours growing in the liver and bone shows sTn is expressed in 37.5% of cases. Furthermore, sTn is expressed in nearly half of the PDX models tested and was found to be broadly androgen‐regulated. These findings identify sTn as a potential prognostic biomarker and therapeutic target in prostate cancer and lay the groundwork for the development of sTn‐targeted precision therapies for advanced disease.

## Graphical abstract

This study utilises a novel antibody (L2A5) to comprehensively monitor sialyl‐Tn antigen (sTn) expression levels in clinical prostate cancer tissues. We reveal sTn is expressed in 44% of prostate tumours and find prostate cancer patients with high sTn levels have significantly poorer survival times. Our findings identify sTn as a potential prognostic biomarker and therapeutic target in prostate cancer and provide a rationale for the development of sTn‐targeted precision therapies for advanced disease.

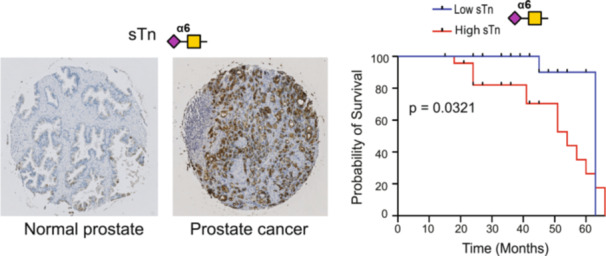

## 1 Introduction

Prostate cancer is a common cancer in males, leading to more than 350,000 deaths worldwide every year [1]. Androgens are required for normal prostate development and function; however, in prostate cancer, the androgen receptor (AR) signalling axis is hijacked to promote disease progression [2]. Drugs that block the production of androgens and/or inhibit AR activity are the cornerstone treatment for advanced prostate cancer, and new AR‐targeted therapies have improved patient outcomes [3, 4, 5]. However, patients still inevitably develop resistance to hormonal therapy, known as castration resistant prostate cancer (CRPC) [6], which remains lethal with a median overall survival ranging from 2 to 3 years [7, 8]. Additional therapeutic strategies are urgently needed to improve outcomes for prostate cancer patients with advanced disease.

Aberrant glycosylation is a hallmark of cancer [9], and in prostate cancer, changes to glycans have been functionally linked to disease progression [10]. *O*‐glycans are found on ~80% of proteins travelling through the secretory pathway and represent one of the most abundant and diverse glycan types [8]. In tumour cells, the processing of *O*‐glycans into branched structures can be disrupted, leading to the expression of the cancer‐associated sialyl‐Tn (sTn) antigen, which is a truncated *O*‐glycan containing a sialic acid α−2,6 linked to GalNAc on serine or threonine residues on glycoproteins. sTn has negligible expression in healthy tissues but is detected in many cancers, such as bladder, ovarian, colon, breast, and pancreatic cancers [11, 12, 13, 14, 15, 16, 17, 18], where it is associated with poor patient prognosis [19, 20]. Mechanistically, sTn plays a role in promoting tumour progression, metastasis, immune evasion, and therapy resistance, and therefore represents an attractive target for the development of anti‐cancer strategies [21, 22]. Over the years, numerous anti‐sTn monoclonal antibodies have been developed, including B72.3, HB‐Stn1, CC49, TKH2, and 3F1 with varying fine specificities [14, 23, 24, 25, 26, 27, 28]. However, anti‐glycan monoclonal antibodies often have low affinity, poor selectivity, and mixed specificity [28, 29, 30]. Recently, a novel monoclonal antibody, known as L2A5, was developed using hybridoma technology that is highly specific to the sTn antigen [31]. L2A5 shows a unique binding pattern specific to invasive regions and metastatic sites in tumours that other anti‐sTn antibodies fail to detect [31, 32]. Notably, L2A5 exhibits fine specificity towards cancer‐associated MUC1 and MUC4 mucin‐derived glycopeptides [32], potentially enabling selective targeting of tumour cells over healthy cells beyond the recognition of the sTn antigen alone [31]. Preclinically, the L2A5 antibody has also been investigated in Chimeric Antigen Receptor (CAR) T‐cell therapy for treatment of STn‐expressing cancers [33, 34]. In triple negative breast cancer (TNBC) patient tissues, L2A5 binds with 23.8% of cases and correlates with significantly reduced survival times in patients, lower c‐Myc expression, and an immunosuppressive tumour microenvironment [17].

In prostate cancer, historical data suggest sTn is expressed in up to 80% of high‐grade prostate tumours [26, 35, 36]. However, these studies were carried out more than 30 years ago, involved limited clinical samples, and utilised anti‐sTn antibodies with now‐recognised limitations of specificity and cross‐reactivity [14, 23, 24, 25, 26, 27, 28]. To date, a comprehensive analysis of sTn across the full clinical spectrum of prostate tumours has not yet been reported and remains a critical research gap in the field [37]. Here, we address this knowledge gap by studying sTn expression in normal prostate tissues, benign prostate hyperplasia (BPH) tissues, primary prostate tumours from both White and Black patients, metastatic CRPC tumour tissues, and in PDX samples. Recognising the limitations of older anti‐sTn antibodies, we chose to utilise the newly developed L2A5 anti‐sTn antibody, which is validated to outperform commonly used anti‐sTn antibodies [31, 32]. Our findings show sTn expression is upregulated in prostate cancer, with sTn specifically detected in prostate tumour cells, and negligible levels detected in normal prostate tissues. We find that high levels of sTn correlate with significantly reduced survival times in prostate cancer patients, and that sTn is expressed in 37.5% of therapy‐resistant metastatic prostate tumours, including prostate‐derived cancers that have spread to the liver and bone. Furthermore, sTn is expressed in prostate cancer patient‐derived xenografts (PDXs), and is broadly androgen‐regulated, identifying a potential tool for evaluating therapeutic approaches to target sTn for prostate cancer.

## 2 Methods

### 2.1 L2A5 Antibody

The monoclonal antibody L2A5 is a proprietary anti‐sTn monoclonal antibody and has been previously described (PCT: WO2019147152A1) [31].

### 2.2 Clinical Samples

#### 2.2.1 TMA Cohort 1

A 96‐case TMA comprising cores of normal prostate tissue and prostate adenocarcinoma of different Grade Groups was purchased from US Biomax (PRC1921‐L38). This TMA includes 5 µm thick, 1.5 mm diameter duplicate cores per case, with cores were obtained from areas with the highest Grade Group and is an updated version of US Biomax TMA PR1921b, which has been previously published by us [38, 39].

#### 2.2.2 TMA Cohort 2

A 100‐case prostate cancer and benign prostate tissue TMA with survival data was purchased from US Biomax (MPR1005sur). This TMA includes 5 µm thick, 1.5 mm diameter single cores per case, with cores were obtained from areas with the highest Grade Group and have been previously published by us and others [40, 41].

#### 2.2.3 TMA Cohort 3

The CHTN_PrC_Prog1 TMA, containing prostate tumour samples from 7 Black and 23 White prostate cancer patients,was obtained through the Cooperative Human Tissue Network (CHTN) at the University of Virginia. This TMA includes 4 µm thick, 1.5 mm‐diameter single cores per case and has been previously published [42].

#### 2.2.4 Metastasis Tissue Samples from Rapid Autopsy (Tissue Cohort 4)

40 cases of rapid autopsy FFPE tissue samples from prostate‐derived tumours growing in bone or liver were kindly provided by Dr Colm Morrissey (University of Washington). Biopsies of metastatic sites were obtained from patients with CRPC within hours of death using a cordless drilling trephine (DeWalt Industrial Tool), model 2422‐51‐000 trephine (DePuy). Liver cores were fixed in 10% neutral buffered formalin and paraffin‐embedded. Bone cores were fixed in 10% neutral buffered formalin, decalcified with 10% formic acid, and paraffin‐embedded. The metastasis tissue samples were full slide sections and were 4 µm thick. The 20 rapid autopsy bone metastasis tissues have been previously published by us [39]. The 20 rapid visceral metastasis tissues are unique to this study and were obtained under ethical approval from the Prostate Cancer Biorepository Network (PCBN) (ref: 210203.1).

### 2.3 LuCaP Patient‐Derived Xenograft (PDX) TMA

The LuCaP PDX TMA contains 39 previously published PDX model tumours [43, 44], established from distinct patients from specimens acquired at either radical prostatectomy or at autopsy, implanted and grown subcutaneously (in triplicate) in intact or castrated mice. Castration resistant derivatives of each model were created by enabling the tumours to grow and progress in castrated mice. The LuCaP models used in our study have been extensively published elsewhere [43, 44, 45, 46, 47, 48, 49, 50, 51, 52, 53], and we refer the reader to these publications for further details regarding each of the models tested. Strict core selection protocols were applied to ensure data quality and exclude areas of necrosis, with cores selectively punched from viable, highly cellular tumour regions, and areas showing necrosis, apoptosis, or mouse stromal contamination were actively avoided. Cores were 5 µm thick, 1.5 mm‐diameter, and included in triplicate per LuCaP line.

### 2.4 Immunohistochemistry

**Figure 1:**
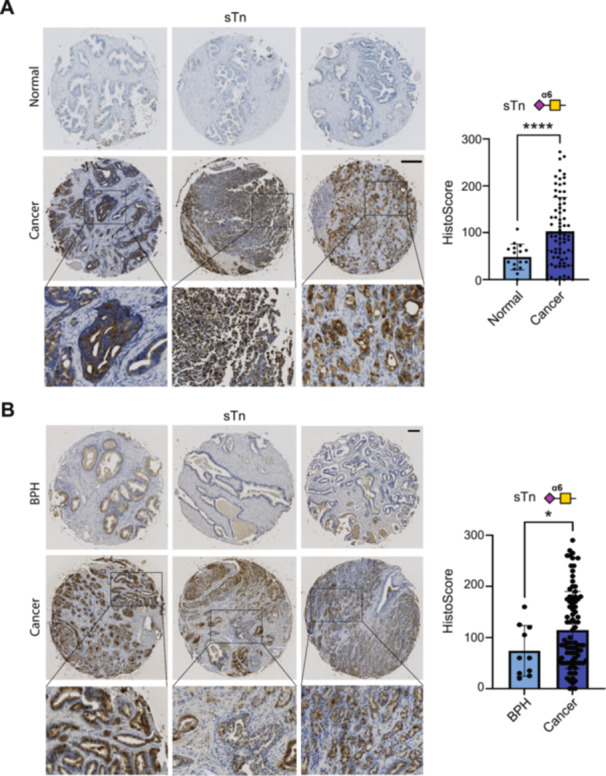
The sTn antigen is expressed in prostate tumours. (A) Analysis of sTn using L2A5 immunohistochemistry in a 96‐case tissue microarray (TMA) comprising 17 normal prostate tissue samples and 79 samples of prostate tumour tissue (TMA cohort 1) showed that sTn is detected at low or negligible levels in normal prostate tissue and is significantly upregulated in prostate tumour tissues (unpaired *t* test, *p* < 0.0001). Furthermore, sTn was detected in 44% of prostate cancer cases at high levels (Histoscore > 100) and at high levels in 15% of cases (Histoscore > 200). (B) L2A5 immunohistochemistry analysis of sTn expression in a previously published 100‐case TMA [40] containing 10 benign prostate hyperplasia (BPH) tissue samples and 90 prostate cancer tissue samples (TMA cohort 2). sTn was detected at significantly higher levels in prostate cancer tissues compared to BPH tissue (unpaired *t*‐test, *p* = 0.0156), with sTn detected in 46% of prostate cancer cases (Histoscore > 100) and at a high level in 23% of cases (Histoscore > 200). Scale bar is 200 µm.

**Figure 2:**
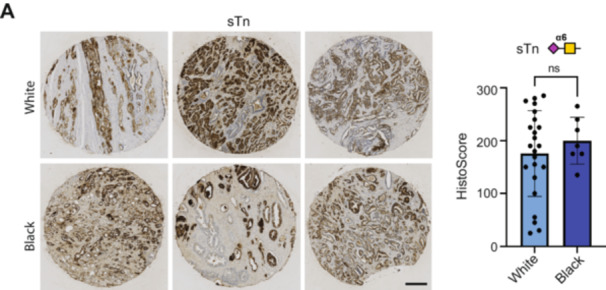
sTn is expressed in Black prostate cancer. L2A5 immunohistochemistry analysis of a previously published TMA [42] comprising prostate tumour samples from 7 Black patients and 23 White patients (TMA cohort 3). No significant differences in sTn levels were detected in prostate tumours from White and Black patients (*n* = 30, unpaired t test, *p* = 0.4603).

**Figure 3:**
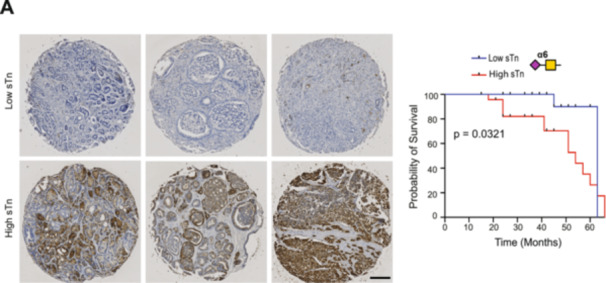
sTn levels correlate with poor patient prognosis in prostate cancer patients. Additional analysis of sTn expression in a 100‐case prostate cancer TMA using L2A5 immunohistochemistry (TMA cohort 2). Stratification of patients based on sTn negative expression (sTn Histoscore < 50) and high sTn expression (sTn Histoscore > 200). Prostate cancer patients with high sTn expression had significantly poorer survival rates compared to patients with negative sTn levels (defined as the bottom 25th percentile of expression) (*n* = 90, Kaplan–Meier regression model, *p* = 0.0321). Scale bar is 200 µm.

IHC was performed with the IHC Prep & Detect Kit for Rabbit/Mouse Primary Antibody (Proteintech, PK10019) following the manufacturer's protocol, with the following exceptions. Slides were dewaxed in Histo‐Clear (SLS, NAT1330) and antigen retrieval was performed with Sodium Citrate antigen retrieval buffer (Proteintech, PR30001). Slides were incubated with 2.5 µg/mL anti‐STn monoclonal antibody L2A5 overnight at 4°C. Coverslips were mounted with Histo‐Mount (SLS, NAT1308) and images were acquired with the ZEISS Axioscan 7 Slide Scanner at 20X magnification. Images were processed using OMERO Plus (Glencoe Software). The TMAs shown in Figures 1, 2, 3 were scored by a pathologist using the 0–300 HistoScore method as described previously [40, 54, 55]. Only epithelial cells were scored. For the PDX TMA shown in Figure 5, images were analysed using QuPath (version 0.6) software (each PDX sample was analysed in triplicate samples from the same mouse, and across three independent mice). Positive cells were outlined and classified as having low, moderate or high staining intensity to calculate a 0‐300 HistoScore, calculated as the sum of each staining intensity score (1+, 2+, 3+) multiplied by the percentage of cells classified at that intensity using the equation staining Index (*H*‐score) = Σ (each intensity score × % of cells at that intensity). In line with previous studies, only epithelial cells were scored. A Histoscore < 50 was considered negative, scores of 50–100 were weakly positive/negligible, scores > 100 were recorded as positive, and scores of > 200 were recoded as highly positive [56, 57].

### 2.5 Statistical Analysis

Statistical analyses were conducted using the GraphPad Prism software (version Prism 10) using suitable tests as described in the legends. Data are presented as the mean of three independent samples ± standard error of the mean (SEM). Statistical significance is denoted as *p < 0.05, **p < 0.01, ***p < 0.001 and ****p < 0.0001.

### 2.6 Study Approval

All human studies were reviewed by the appropriate ethics committee and performed in accordance with the ethical standards laid down in the Declaration of Helsinki. All autopsy tissues were collected from patients who had signed written informed consent under the aegis of the Prostate Cancer Donor Program at the University of Washington [44]. The IRB of the University of Washington approved this study. All patient‐derived xenograft experiments were approved by the University of Washington IACUC. For animal experiments, the ‘Principles of Laboratory Animal Care’ were followed as well as specific national laws as detailed in previous publications [43, 44].

## 3 Results

### 3.1 The Cancer‐Associated sTn Antigen is Upregulated in Prostate Tumours

Previous studies suggest that the sTn antigen is expressed in prostate cancer tissues [26, 35, 36]. To overcome the limitations of the anti‐sTn antibodies used in prior studies, we utilised the L2A5 anti‐sTn monoclonal antibody, which has stood out for its reported specificity towards sTn‐expressing tumour tissues [31]. Using L2A5 immunohistochemistry, we monitored the levels of sTn in three distinct patient cohorts. In a 96‐case tissue microarray (TMA) containing 17 normal prostate tissue samples and 79 samples of prostate tumour tissue, sTn was significantly higher in prostate tumour tissues compared to normal prostate tissues (*p* < 0.001) (Figure 1A). sTn was expressed in 44% prostate cancer tissues tested (sTn Histoscore > 100), with high expression detected in 15% of cases (sTn Histoscore > 200). sTn was absent in 28% of prostate cancer tissues (sTn Histoscore < 50), with a further 28% of cases having negligible levels of sTn (sTn Histoscore 50–100). sTn was absent or detected at negligible levels in 100% of normal prostate tissues (sTn Histoscore < 100). Further analysis of a 100 case TMA, containing 10 benign prostate hyperplasia (BPH) tissue samples and 90 prostate cancer tissues, revealed sTn was expressed in 46% of prostate tumours (sTn Histoscore > 100), with 23% of cases having high sTn expression (sTn Histoscore > 200), and levels significantly higher in prostate tumours compared to BPH samples (*p* = 0.0156) (Figure 1B). Consistent with previous studies in breast cancer tissues, all sTn‐positive cases in our cohorts showed membrane staining and additional cytoplasmic signal, which is likely a reflection of the biosynthetic pathway of sTn [17]. Our findings, using a well‐validated, highly specific antibody, show the cancer‐associated sTn antigen is significantly upregulated in prostate tumours compared to normal or benign prostate tissues.

### 3.2 Expression of sTn in Black Prostate Cancer

The data presented above reveals sTn is expressed in prostate tumour tissues. As Black patients with prostate cancer have higher incidence rates, worse outcomes, and differences in tumour biology [58], we next investigated if the levels of the sTn antigen might be altered in Black prostate cancer. Analysis of sTn levels in a TMA comprising 7 Black prostate cancer tissues and 23 White prostate cancer tissues revealed sTn is expressed in 6/6 (100%) of Black tumour tissues and 20/24 (83%) of White tumour tissues (sTn HistoScore > 100). Overall, we detected no significant difference in sTn levels between Black and White prostate cancer (*n* = 30, unpaired *t* test, *p* = 0.4603) (Figure 2). Taken together, these findings show the sTn antigen may be expressed at similar levels in White and Black prostate tumour tissues, and that once anti‐sTn therapies become available for cancer therapy, they will likely be relevant to both White and Black patients with prostate cancer.

### 3.3 High sTn Expression Correlates With Reduced Survival Times in Prostate Cancer Patients

Next, to investigate the prognostic and therapeutic potential of sTn in prostate cancer, we further analysed the association between sTn expression and clinical parameters. As sTn correlates with poor patient prognosis in breast cancer [17], we tested if expression of sTn might also correlate with survival times in prostate cancer patients. We analysed a previously published TMA comprising 90 cases of primary prostate cancer tissues (TMA cohort 2) [41]. Here, when we stratified prostate cancer patients based on sTn negative and sTn high levels (defined as Histoscore < 50 compared to Histoscore > 200), patients with high sTn levels had significantly reduced survival rates compared to patients with negative sTn levels (*p* = 0.0321) (Figure 3). Our data shows high sTn levels correlate with poorer patient prognosis in prostate cancer and provides a rationale for future studies investigating anti‐sTn therapeutic strategies for prostate cancer.

### 3.4 sTn is Expressed in Metastatic Therapy Resistant Prostate Cancer

**Figure 4:**
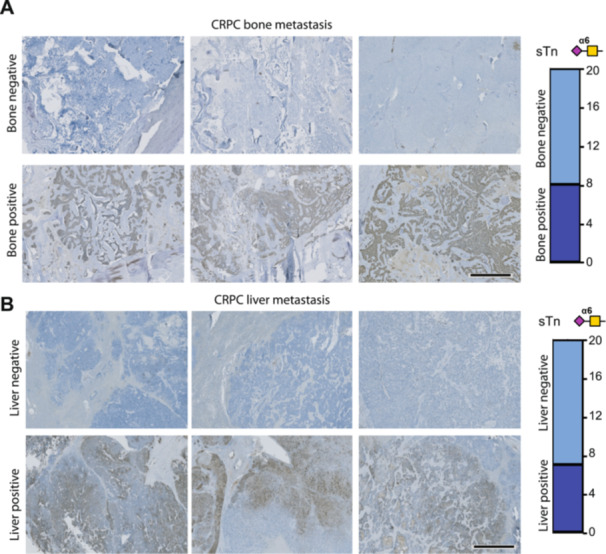
sTn is expressed in lethal metastatic therapy‐resistant prostate cancer. Analysis of sTn levels in 40 metastatic CRPC tumours obtained via rapid autopsy (tissue cohort 4). (A) L2A5 immunohistochemistry shows sTn is expressed by 8/20 prostate‐derived tumours growing in bone, and (B) 7/20 prostate‐derived tumours growing in soft tissue (liver). Scale bar is 2000 µm.

Nearly all men with advanced prostate cancer who receive hormone therapy eventually progress to CRPC, where metastasis is common [8]. Although previous studies have analysed the levels of glycosyltransferase enzymes and changes to *N*‐glycans in CRPC [39, 59, 60], the expression levels of sTn in metastatic therapy‐resistant disease has not yet been evaluated. To address this research gap, we next monitored sTn in 40 rapid autopsy tissues obtained from lethal prostate‐derived liver and bone metastatic tumours. L2A5 immunohistochemistry showed sTn was detected in 15/40 CRPC samples tested, including 8/20 bone tumours (Figure 4A) and 7/20 liver tumour tissues (Figure 4B). Our findings suggest sTn is highly expressed in 37.5% lethal metastatic prostate tumours and identify the sTn antigen as a potential therapeutic target for the development of new therapies for advanced disease.

### 3.5 sTn Expression Validation in Prostate Cancer Patient‐Derived Xenografts (PDXs)

**Figure 5:**
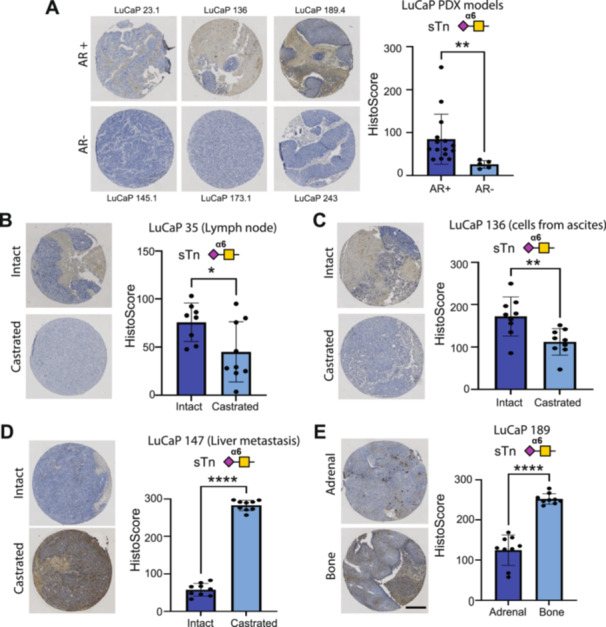
Analysis of sTn in LuCaP patient‐derived xenografts (PDX) tissues. L2A5 immunohistochemistry was used to analyse the sTn antigen in tissue samples from the LuCaP PDX series [43]. (A) Analysis of sTn Histoscores showed a significant decrease in sTn levels in AR‐null (neuroendocrine) prostate cancer models compared to AR‐positive prostate cancer models grown in intact mice (*n* = 20, unpaired *t* test, *p* = 0.0017). (B) For LuCaP 35 (established from lymph node metastasis), there was a significant reduction in sTn levels in PDX tissues grown in castrated mice compared to intact mice (*n* = 18, paired *t* test, *p* = 0.0288). (C) For LuCaP 136 (grown from cells from ascites), there was a significant reduction in sTn levels in PDX tissues grown in castrated mice compared to intact mice (n = 18, paired t test, *p* = 0.0061). (D) In LuCaP 147, which was established from implanted tissue from liver metastasis, the levels of sTn are significantly increased in PDX samples grown in castrated conditions compared to intact mice (*n* = 6, paired *t* test, *p* < 0.0001). (E) Comparison of sTn levels in PDX tissues established from adrenal metastasis (LuCaP 189.3) and bone metastasis tissue (LuCaP 189.4) from the same patient shows sTn is significantly increased in PDX samples established from bone metastatic tissue (*n* = 6, paired t test, *p* = 0.0168). Representative images for PDX models are shown. Each PDX sample was analysed in triplicate within 3 mice. Scale bar is 200 µm.

Next, with the longer‐term goal of identifying a clinically relevant platform for the future evaluation of anti‐sTn therapeutic for prostate cancer, we assessed sTn expression within the LuCaP prostate cancer PDX series, which reflects the diverse molecular composition of human CRPC [43, 44]. Using L2A5 immunohistochemistry, we evaluated 39 PDXs, representing a spectrum of metastatic sites and histologies, grown in both intact and castrated immunodeficient mice. Among the 39 PDXs evaluated, after grafting patient tumour cells into a new microenvironment, 19 showed positive sTn expression, and 20 PDXs tested showed no sTn expression. Some sTn‐positive samples showed moderate sTn staining, while others displayed strong positive staining for sTn, with sTn‐positive staining detected in PDXs established from ascites, and from liver and bone metastatic tissues (Figure 5). To assess if sTn levels might change with AR status, we compared AR‐positive (adenocarcinoma) and AR‐null (neuroendocrine) LuCaP models. This revealed that sTn levels are significantly lower in AR‐null samples compared to LuCaP models that are AR‐positive, with all five AR‐null models testing negative for sTn (Histoscore < 50) (Figure 5A). Next, to further test for androgen regulation of sTn, we selected LuCaP 35, LuCaP 136, and LuCaP 147 as highly androgen‐sensitive prostate cancer models [46, 47, 51]. For both LuCaP 35 and LuCaP 136, tumours grown in castrated conditions had significantly lower levels of sTn compared to tumours grown in intact mi (Figure 5B,C). Conversely, LuCaP 147 demonstrated a significant increase in sTn expression in castrated conditions (Figure 5D). Interestingly, in matched PDX tissues from the same patient (LuCaP 189), sTn levels were significantly increased in PDX models established from a bone tumour compared to those from an adrenal tumour (Figure 5E). Taken together, our data show that LuCaP PDX models retain sTn expression as a key feature of patient tumours and that sTn exhibits broad androgen regulation in these models, supporting their utility for accelerating anti‐sTn therapeutic development.

## 4 Discussion

The truncated *O*‐glycan structure sTn has emerged as a compelling target for cancer therapeutics due to its aberrant yet specific expression pattern on epithelial tumours. While detection of sTn is rare or absent in normal tissues, sTn is prominently expressed in various epithelial tumours, and targeting sTn holds tremendous potential to treat a wide range of solid tumours. In prostate cancer, historical studies have detected sTn in up to 50%–80% of prostate tumours [25, 30, 31]. However, these studies were based on limited sample sizes and utilised benchmark antibodies that now have recognised limitations. For example, B72.3 shows a clear preference for serine over threonine residues [61, 62] and retains around 26% of binding to sTn‐positive cells treated with sialidase [31]. L2A5 also preferentially binds serine over threonine residues, but L2A5 binding drops to negligible/undetectable levels following sialidase digestion on sTn‐positive cancer cells, highlighting its superior fine specificity [31]. Given the major importance held by the accurate detection of sTn in prostate cancer for advancing the development of targeted therapies, we utilised the L2A5 anti‐sTn antibody, which overcomes key limitations of previous anti‐sTn antibodies, to monitor the sTn antigen in prostate tumour tissues. Our study provides a novel and comprehensive characterisation of the sTn antigen in prostate cancer tissues representing the full clinical spectrum of the disease. We report 44% of prostate tumours express sTn with selective staining of malignant tissues and limited reactivity toward normal prostate tissue. Consistent with previous studies in other cancer types [17, 63, 64], high sTn levels correlate with significantly poorer survival outcomes in prostate cancer patients. Furthermore, we show sTn remains expressed in lethal therapy‐resistant prostate tumours obtained via rapid autopsy and in clinically relevant CRPC PDX models. Our findings underscore the promise and provide a rationale for future pre‐clinical studies exploring the development of sTn‐targeted therapies for prostate cancer.

Previous studies have suggested that both ST6 N‐acetylgalactosaminide alpha‐2,6‐sialyltransferase 1 (ST6GalNAc1), the primary enzyme responsible for sTn synthesis [65], and sTn itself are regulated by androgens in prostate cancer cells [66, 67]. Here, we monitored sTn levels in the LuCaP PDX series, which includes both AR‐positive and AR‐null (neuroendocrine) phenotypes, as well as paired models representing androgen‐sensitive and castration‐resistant variants. Consistent with the androgen‐dependent regulation of sTn in prostate cancer, we found that sTn levels correlate with AR status across LuCaP models. Furthermore, in two well‐established androgen‐responsive models, LuCaP 35 and LuCaP 136 [46, 50], sTn levels decreased significantly in castrated samples. In contrast, in LuCaP 147 [51], sTn levels increased significantly in tumours grown in castrated mice. Notably, LuCaP 147 is poorly differentiated and harbours both an SPOP mutation and a mismatch repair hypermutator phenotype [51]. We hypothesise that the castration‐dependent upregulation of sTn in the LNCaP 147 model may reflect adaptive tumour evolution, whereby androgen signalling is maintained or reactivated despite low circulating androgen levels, leading to context‐dependent regulation of sTn. Consistent with this, sTn was also detected in metastatic CRPC tissue samples, supporting its persistence in advanced disease. Taken together, these findings suggest that sTn levels are associated with androgen signalling in prostate cancer cells, although this relationship appears to be context dependent, reflecting the molecular heterogeneity of prostate cancer biology.

The sTn antigen is a recognised mediator of immune evasion and an immunosuppressive tumour microenvironment [22, 68]. sTn interacts with immune cells through lectins such as Siglecs and MGL to promote tumour immune suppression [69, 70]. In bladder cancer, sTn impairs dendritic cell maturation and suppresses anti‐tumour T cell responses [22], while in breast cancer it is associated with M2 macrophage polarization [17]. Although the role of sTn in prostate cancer remains unclear, Siglec‐7, −9, −10, and −15, which can bind sTn [21, 71, 72, 73, 74], are expressed by immunosuppressive macrophages and NK cells in the prostate tumour microenvironment [40, 75, 76]. Together, these findings suggest that sTn may contribute to immune suppression in prostate cancer and support further investigation into the Siglec–sTn and MGL–sTn axes in disease progression.

The expression of sTn on the surface of cancer cells makes it a promising target for cancer therapy, particularly as healthy adult cells do not express sTn, which will prevent off‐target toxicity. The Theratope vaccine, which targets sTn, was evaluated in Phase I and II clinical trials, and succeeded in triggering T cell‐dependent responses against cancer cells in breast and ovarian cancer patients [77], but failed to show improved overall survival in phase III clinical trials [78]; potentially as the trial did not stratify/select sTn+ patients for inclusion which later studies indicate is important [79]. Additional strategies being explored to block or target sTn include CAR and antibody‐based approaches. Antibodies targeting sTn have emerged with different specificities against the epitope [27, 31, 62, 80, 81]. Targeted therapy based on antibodies is a rapidly expanding field, with > 150 monoclonal antibodies receiving FDA approval in the last 40 years [82], and antibodies targeting sTn hold promise to enhance immune responses against sTn‐positive tumours. The monoclonal antibody 3P9 has been reported to directly inhibit the growth of colorectal tumours [81], and an sTn antibody‐drug conjugate (ADC) has demonstrated efficacy against sTn‐expressing tumours in vivo [25]. The clinical potential of anti‐sTn antibody‐drug conjugates is demonstrated by SGN‐STNV, which was evaluated in a clinical trial for solid tumours (NCT04665921). Furthermore, anti‐STn CAR‐T cell therapies have shown significant anti‐cancer activity in pre‐clinical mouse models [33, 34, 62], and anti‐TAG72 antibodies that target mucin‐enriched sTn are being investigated in a clinical trial for the treatment of ovarian cancer (NCT05225363).

This study has several limitations that should be considered. First, as a descriptive study, the findings demonstrate associations between sTn expression, disease state, and androgen signalling, but do not investigate the underlying mechanisms or functional consequences. In addition, while the use of multiple prostate cancer cohorts and LuCaP PDX models strengthens the robustness of the study, TMAs contain only small tumour cores and may not fully capture intratumoral heterogeneity. Consequently, correlations between sTn levels and Grade Groups were not assessed. Similarly, although PDX models represent clinically relevant prostate cancer phenotypes, they do not completely recapitulate the complexity of the human tumour microenvironment. Finally, metastatic CRPC was represented by relatively small sample numbers, which may limit our findings. Further studies using larger tissue sections and functional approaches will therefore be important to better define the biological and clinical significance of sTn in prostate cancer progression.

In conclusion, here we present a comprehensive analysis of the cancer‐associated sTn antigen in prostate cancer tissues representing both untreated primary disease as well as therapy‐resistant metastatic tumours. Using a novel anti‐sTn antibody (L2A5) with unique binding specificity to sTn, we show that the sTn antigen is upregulated in prostate tumour tissue and correlates with poorer survival rates in patients. Furthermore, sTn remains expressed in metastatic CRPC. Our findings introduce the sTn glycan epitope as a promising therapeutic target in prostate cancer and identify the LuCaP series of PDXs [43] as a pre‐clinical platform for the future testing of anti‐sTn agents.

## Author Contributions

K.H., E.S., A.S., M.O.M., and Z.P. performed immunohistochemistry optimisation, validation, and analysis of clinical tissues. L.B. performed data analysis. P.V. provided the L2A5 antibody for the study. J.M., K.H., Z.P., E.S., L.B., and M.O.M. verified the underlying data. J.M., K.H., and P.V. jointly designed, analysed, and interpreted the study. J.M. and K.H. wrote the original manuscript draft and made the figures. J.M., P.V., K.H., E.S., M.O.M., Z.P., A.S., and R.B. contributed to the critical review of the manuscript. J.M., K.H., and R.B. contributed to funding acquisition. K.H., J.M., M.O.M., and R.B. contributed to project supervision. All authors read the manuscript, agreed with the content, and were given the opportunity to provide input.

## Ethics Statement

The prostate cancer tissue samples analysed in Figures 3 and 4 were kindly provided by the Prostate Cancer Biorepository Network (PCBN). Written informed consent was obtained from all patients. The PCBN ethics committee reviewed our project and provided ethical approval for the use of the samples in our project (ref: 210203.1).

## Conflicts of Interest

P.A.V. was co‐founder of CellmAbs and inventor on patent WO2019147152A1. All other authors have no conflicts of interest to declare.

## Data Availability

The data that support the findings of this study are available from the corresponding author upon reasonable request.
